# Patients’ characteristics and mortality in urgent/emergent/salvage transcatheter aortic valve replacement: insight from the OCEAN-TAVI registry

**DOI:** 10.1136/openhrt-2020-001467

**Published:** 2020-12-14

**Authors:** Yusuke Enta, Masaki Miyasaka, Masataka Taguri, Norio Tada, Masaki Hata, Yusuke Watanabe, Toru Naganuma, Masahiro Yamawaki, Futoshi Yamanaka, Shinichi Shirai, Hiroshi Ueno, Kazuki Mizutani, Minoru Tabata, Kensuke Takagi, Masanori Yamamoto, Kentaro Hayashida, OCEAN-TAVI investigators

**Affiliations:** 1 Cardiovascular Center, Sendai Kousei Hospital, Sendai, Miyagi, Japan; 2 Department of Biostatistics, Yokohama City University School of Medicine, Yokohama, Kanagawa, Japan; 3 Cardiology, Teikyo University School of Medicine, Itabashi-ku, Tokyo, Japan; 4 Cardiology, New Tokyo Hospital, Matsudo, Chiba, Japan; 5 Cardiology, Saiseikai Yokohama-City Eastern Hospital, Yokohama, Kanagawa, Japan; 6 Cardiology, Shonan Kamakura General Hospital, Kamakura, Kanagawa, Japan; 7 Cardiology, Kokura Memorial Hospital, Kitakyusyu, Fukuoka, Japan; 8 Cardiology, Toyama University Hospital, Toyama, Toyama, Japan; 9 Cardiovascular Medicine, Osaka City General Hospital, Osaka, Osaka, Japan; 10 Cardiovascular Surgery, Tokyo Bay Urayasu Ichikawa Medical Center, Urayasu, Chiba, Japan; 11 Cardiology, Ogaki Municipal Hospital, Ogaki, Gifu, Japan; 12 Cardiology, Toyohashi Heart Center, Toyohashi, Aichi, Japan; 13 Cardiology, Nagoya Heart Center, Nagoya, Aichi, Japan; 14 Cardiology, Keio University School of Medicine, Shinjuku-ku, Tokyo, Japan

**Keywords:** transcatheter aortic valve replacement, aortic valve stenosis, heart valve prosthesis implantation

## Abstract

**Objectives:**

Patients’ backgrounds and clinical outcomes in urgent/emergent/salvage transcatheter aortic valve replacement (Em-TAVR) remain unclear. We investigated patient characteristics and the mortality in Em-TAVR and the predictors for the need for Em-TAVR.

**Methods:**

We consecutively enrolled 1613 patients undergoing TAVR for severe aortic stenosis between October 2013 and July 2016 from the Optimised transCathEter vAlvular interventioN (OCEAN)-transcatheter aortic valve implantation (TAVI) registry. The urgency was based on the European System for Cardiac Operative Risk Evaluation II. Urgent, emergent or salvage were included with the Em-TAVR group and elective with the El-TAVR group.

**Results:**

Em-TAVR was observed in 87 (5.4%) patients. A higher Clinical Frailty Scale (CFS), peripheral artery disease (PAD), hypoalbuminaemia, reduced left ventricular ejection fraction (LVEF) and preoperative at least moderate mitral regurgitation (MR) predicted the need for the Em-TAVR by the multivariate logistic regression analysis. The Em-TAVR group had the higher Society of Thoracic Surgeons Score (13.7 (IQR 8.2–21.0) vs 6.5 (IQR 4.6–9.2); p<0.001) and higher 30-day mortality (9.2% vs 1.3%; p<0.001) than the El-TAVR group. Accordingly, Kaplan-Meier analysis showed that the cumulative mortality was higher in the Em-TAVR group than that in the El-TAVR group (log-rank; p<0.001). However, Em-TAVR did not predict mortality in the multivariate Cox regression analysis.

**Conclusions:**

Em-TAVR was performed in 5.4% of patients. Higher CFS, PAD, hypoalbuminaemia, reduced LVEF and preprocedural MR predicted the need for Em-TAVR. Em-TAVR was not a predictor for mortality in the multivariate analysis, suggesting that it is a reasonable treatment option.

**Key questions**

**What is already known about this subject?**

- Previous studies suggested urgent/emergent/salvage transcatheter aortic valve replacement (Em-TAVR) as an effective treatment option in patients with severe aortic stenosis (AS) with acute decompensated heart failure. However, because the backgrounds of patients who underwent Em-TAVR differed across these studies, the effectiveness and safety of Em-TAVR have not been confirmed and predictors of need for Em-TAVR remain unclear.

**What does this study add?**

- The percentage of patients with severe AS who needed to undergo Em-TAVR in our study was 5.4%. The predictors for the need for Em-TAVR were a high Clinical Frailty Scale, a history of peripheral artery disease, hypoalbuminaemia, reduced left ventricular ejection fraction and at least moderate mitral regurgitation. Urgency did not negatively affect the mortality after TAVR according to the multivariate analysis. This is the first report on Em-TAVR from an Asian multicentre registry.

**How might this impact on clinical practice?**

- As the finding that Em-TAVR itself does not predict mortality, there may be no need for hesitation when deciding to perform Em-TAVR. It may be better to consider the procedure ahead of time in patients with severe AS with congestive heart failure, especially in those using catecholamines and/or mechanical circulatory support because bed rest is associated with sarcopenia, infections and a greater length of stay.

## Introduction

Transcatheter aortic valve replacement (TAVR) has emerged as a safe and effective treatment option for patients with symptomatic severe aortic stenosis (AS) who are at prohibitive, high or intermediate risk for surgical aortic valve replacement (SAVR).^1^ Recently, the U.S. Food and Drug Administration approved an expanded indication for several transcatheter heart valves to include patients with severe AS at low surgical risk.^2 3^

Previous studies suggested urgent/emergent/salvage TAVR (Em-TAVR) as an effective treatment option in patients with severe AS with acute decompensated heart failure or cardiogenic shock.^4–7^ However, the baseline characteristics of patients who underwent emergent TAVR differed across these studies. For example, regarding a status of urgency of TAVR procedure, no ‘salvage’ status patient, ‘emergent’ with 0.2% of patients and ‘urgent’ with 9.7% were included in the study from the Society of Thoracic Surgeons (STS) and the American College of Cardiology Transcatheter Valve Therapy (ACC TVT) Registry.^4^ In contrast, another study only included patients with cardiogenic shock.^5^ Thus, the results of these previous studies should be interpreted with caution. The patients’ backgrounds and clinical outcomes in Em-TAVR have not been thoroughly studied.

The aim of this study was to investigate the predictors for the need for Em-TAVR, and the patients’ characteristics and mortality in Em-TAVR using data from a multicentre Japanese registry.

## Methods

### Study population and definitions

The Optimised transCathEter vAlvular interventioN TAVI (OCEAN-TAVI) registry is a Japanese multicentre prospective registry affiliated to 14 high-volume medical centres, including the Keio University School of Medicine, Teikyo University School of Medicine, New Tokyo Hospital, Kokura Memorial Hospital, Saiseikai Yokohama-City Eastern Hospital, Sendai Kosei Hospital, Shonan Kamakura General Hospital, Toyohashi Heart Center, Nagoya Heart Center, Toyama University, Tokyo bay medical centre, Osaka city university Hospital, Kishiwada tokusyu-kai Hospital and Ogaki Municipal Hospital. This trial is registered with the University Hospital Medical Information Network (UMIN; UMIN000020423). Between October 2013 and July 2016, 1613 patients with severe AS undergoing TAVR with the Edwards Sapien XT and Sapien 3 valve (Edwards Lifesciences) and the Medtronic CoreValve (Medtronic, Minneapolis, Minnesota, USA) were included in the OCEAN-TAVI registry. The inclusion criteria for this registry have been previously reported.^8^

Patients who admitted for a planned TAVR operation were included in the elective TAVR (El-TAVR) group. The others who required unplanned hospitalisation and TAVR during the same hospitalisation were included in the Em-TAVR group. The level of urgency was defined as urgent, emergent or salvage, based on the European System for Cardiac Operative Risk Evaluation (EuroSCORE) II risk model.^9^ Urgent: a status requiring unplanned hospitalisation and TAVR during the same hospital stay due to unstable symptoms, catecholamine dependency and/or a need for mechanical circulatory support (MCS), such as intra-aortic balloon pumping, extracorporeal membrane oxygenation or mechanical respiratory support. Emergent: requiring TAVR before the beginning of the next working day after the decision to operate. Salvage: requiring cardiopulmonary resuscitation (external cardiac massage) before TAVR and subsequent TAVR.

### Statistical analysis

Continuous variables were assessed for normality of distribution using the Shapiro-Wilk test; those that followed a normal distribution were reported as the mean±SD, and those that did not were reported as the median and IQR. Student’s t*-*test was performed for intergroup comparisons of parametric data, and the Mann-Whitney U test was used for intergroup comparisons of non-parametric data. Categorical variables were reported as a number (percentage) and compared using Pearson’s χ^2^ test or Fisher’s exact test. A p<0.05 was considered statistically significant. Parameters for the prediction (p<0.05) of the need for Em-TAVR were entered into a multivariable logistic regression model. To determine independent predictors for all-cause mortality after TAVR, multivariate Cox proportional hazards models were used. Cumulative mortality was estimated by the Kaplan-Meier method, and differences were assessed with the log-rank test. JMP V.14 for Mac (SAS Institute) was used for all statistical analyses.

## Results

### Baseline characteristics and echocardiographic variables

Of the 1613 patients in our study, 87 (5.4%) patients underwent Em-TAVR (70 (4.3%) urgent, 15 (0.9%) emergent and 2 (0.1%) salvage) due to decompensated heart failure. The remaining 1526 patients (94.6%) underwent El-TAVR. The median follow-up time was 250 (IQR 99–447) days. The baseline patient characteristics, laboratory data and echocardiography data are listed in table 1. The mean age was 84.4±5.1 years and 70.4% were female in the all TAVR patients. The Em-TAVR patients had the higher Surgical Risk Scores compared with the El-TAVR patients (STS Score: 13.7 (IQR 8.2–21.0) vs 6.5 (IQR 4.6–9.2); p<0.001 and EuroSCORE II: 11.0 (IQR 4.6–19.2) vs 3.6 (IQR 2.3–5.6); p<0.001). Regarding preoperative echocardiographic findings, Em-TAVR patients had a lower left ventricular ejection fraction (LVEF) than El-TAVR patients (47.9%±16.1% vs 58.5%±11.9%, p<0.001). Compared with El-TAVR patients, Em-TAVR patients had a higher prevalence of at least moderate mitral regurgitation (MR) (27.6% vs 9.0%, p<0.001) and at least moderate tricuspid regurgitation (TR) (14.9% vs 6.0%, p=0.004). Contrast-enhanced CT was performed for all patients in this study.

**Table 1:** Baseline patient characteristics of the study population

Variable	Em-TAVR (n=87 (5.4%))	El-TAVR (n=1526 (94.6%))	P value
Baseline patient characteristic			
Age, years	84.9±7.0	84.3±5.0	0.282
Female, n (%)	61 (70.1)	1075 (70.4)	0.948
Height, cm	149.1±8.2	149.8±9.1	0.509
Weight, kg	47.3±9.3	50.0±10.1	0.018
Body mass index, kg/m^2^	21.3±2.3	22.2±3.6	0.020
Clinical Frailty Scale	5.0±1.3	3.9±1.2	<0.001
NYHA functional class III or IV, n (%)	77 (88.5)	740 (48.5)	<0.001
Prior heart failure, n (%)	81 (93.1)	1232 (80.7)	0.001
Syncope, n (%)	13 (14.9)	173 (11.3)	0.325
Current smoker, n (%)	7 (8.1)	37 (2.4)	0.010
Hypertension, n (%)	65 (74.7)	1203 (78.8)	0.371
Diabetes mellitus, n (%)	29 (33.3)	401 (26.3)	0.157
Dyslipidaemia, n (%)	40 (46.0)	648 (42.5)	0.521
Peripheral artery disease, n (%)	27 (31.0)	219 (14.4)	<0.001
COPD, n (%)	15 (17.4)	282 (18.5)	0.770
Atrial fibrillation, n (%)	31 (35.6)	308 (20.2)	0.001
Prior MI, n (%)	13 (14.9)	103 (6.8)	0.010
Prior PCI, n (%)	27 (31.0)	404 (26.5)	0.357
Prior CABG, n (%)	9 (10.3)	111 (7.2)	0.555
Prior pacemaker implantation, n (%)	8 (9.2)	107 (7.0)	0.459
Prior stroke, n (%)	20 (23.0)	211 (13.8)	0.026
Urgency of procedure			<0.001
Elective, n (%)	0 (0.0)	1526 (100.0)	
Urgent without catecholamine or MCS, n (%)	41 (47.1)		
Urgent with catecholamine or MCS, n (%)	29 (33.3)		
Emergent, n (%)	15 (17.2)		
Salvage, n (%)	2 (2.3)		
STS score, %	13.7 (8.2–21.0)	6.5 (4.6–9.2)	<0.001
Logistic EuroSCORE, %	29.8 (17.4–48.3)	12.6 (7.9–20.5)	<0.001
EuroSCORE II, %	11.0 (4.6–19.2)	3.6 (2.3–5.6)	<0.001
State of catecholamine dependency, n (%)	36 (41.4)	0 (0.0)	<0.001
Use of IABP, n (%)	8 (9.2)	0 (0.0)	<0.001
Laboratory data			
Haemoglobin concentration, g/dL	10.8±1.9	11.2±1.6	0.010
eGFR, (mL/min/1.73 m^2^)	45.5±22.1	52.3±20.1	0.002
Albumin, g/dL	3.4±0.5	3.8±0.5	<0.001
Albumin <3.5 g/dL, n (%)	54 (62.1)	430 (28.2)	<0.001
Brain natriuretic peptide, pg/mL	1200±1357	423±551	<0.001
Preoperative echocardiographic data			
LVEF (modified Simpson), %	47.9±16.1	58.5±11.9	<0.001
Aortic valve area, cm^2^	0.56±0.15	0.64±0.17	<0.001
Index aortic valve area, cm^2^/m^2^	0.41±0.11	0.45±0.12	0.003
Mean pressure gradient, mm Hg	50.2±20.0	50.5±18.0	0.866
Peak velocity, m/s	4.5±0.84	4.6±0.78	0.413
Aortic regurgitation ≥ moderate, n (%)	9 (10.3)	146 (9.6)	0.813
Mitral regurgitation ≥ moderate, n (%)	24 (27.6)	138 (9.0)	<0.001
Tricuspid regurgitation ≥ moderate, n (%)	13 (14.9)	92 (6.0)	0.004

Values are presented as mean±SD unless otherwise stated.

P<0.05 were considered statistically significant.

CABG, coronary artery bypass graft; COPD, chronic obstructive pulmonary disease; eGFR, estimated glomerular filtration rate; El-TAVR, elective transcatheter aortic valve replacement; Em-TAVR, urgent/emergent/salvage transcatheter aortic valve replacement; EuroSCORE, European System for Cardiac Operative Risk Evaluation; IABP, intra-aortic balloon pumping; LVEF, left ventricular ejection fraction; MCS, mechanical circulatory support; MI, myocardial infarction; NYHA, New York Heart Association; PCI, percutaneous coronary intervention; STS, Society of Thoracic Surgeons; TAVR, transcatheter aortic valve replacement.

### Procedural characteristics and in-hospital outcomes

The procedural characteristics and clinical outcomes are presented in table 2. The 30-day mortality in Em-TAVR patients was higher than in El-TAVR patients (9.2% vs 1.3%, p<0.001). Acute device success, according to the Valve Academic Research Consortium 2 definition,^10^ was achieved in 76 (87.4%) Em-TAVR patients and in 1440 (94.4%) El-TAVR patients. Among the patients in our study, the Clinical Frailty Scale (CFS) score (OR 1.50, 95% CI 1.24 to 1.83, p<0.001), peripheral artery disease (PAD; OR 2.34, 95% CI 1.32 to 4.13, p=0.003), serum albumin concentration <3.5 g/dL (OR 2.15, 95% CI 1.25 to 3.68, p=0.006), LVEF (OR 0.96, 95% CI 0.94 to 0.97, p<0.001) and preoperative moderate or severe MR (OR 2.60, 95% CI 1.42 to 4.74, p=0.002) were identified by the multivariate logistic regression analysis as predictors for the need for Em-TAVR (table 3). According to the multivariate Cox regression analysis, TAVR urgency was not associated with mortality after TAVR (online supplemental table 1).

10.1136/openhrt-2020-001467.supp1 Supplementary data

**Table 2:** Procedural characteristics and in-hospital outcomes in transcatheter aortic valve replacement

Variable	Em-TAVR (n=87)	El- TAVR (n=1526)	P value
Procedural characteristics			
Transfemoral approach, n (%)	72 (82.8)	1241 (81.3)	0.736
Bioprosthetic valve type			0.171
Sapien XT, n (%)	70 (80.5)	1258 (82.4)	
Sapien 3, n (%)	12 (13.7)	129 (8.5)	
CoreValve, n (%)	5 (5.8)	139 (9.1)	
Predilatation, n (%)	65 (74.7)	1180 (77.3)	0.576
Postdilatation, n (%)	18 (20.7)	256 (16.8)	0.357
Use of ECMO, n (%)	14 (16.1)	8 (0.5)	<0.001
Elective ECMO, n (%)	10 (11.5)	6 (0.4)	<0.001
Emergent ECMO, n (%)	4 (4.6)	2 (0.1)	<0.001
Contrast volume, mL	120.5±71.1	123.5±59.4	0.659
Fluoroscopic time, min	25.2±12.1	21.2±9.7	<0.001
Clinical outcomes and complications			
30-day mortality, n (%)	8 (9.2)	20 (1.3)	<0.001
In-hospital death, n (%)	10 (11.5)	42 (2.8)	<0.001
Device success, n (%)	76 (87.4)	1440 (94.4)	0.018
Acute coronary obstruction, n (%)	1 (1.1)	12 (0.8)	0.728
New pacemaker implantation, n (%)	8 (9.2)	121 (7.9)	0.681
Stroke, n (%)	3 (3.4)	24 (1.3)	0.231
Life-threatening bleeding, n (%)	13 (14.9)	81 (5.3)	0.001
Major bleeding, n (%)	19 (21.8)	203 (13.3)	0.035
Transfusion, n (%)	47 (54.0)	471 (30.9)	<0.001
Major vascular complication, n (%)	13 (14.9)	76 (5.0)	<0.001
AKI stage 1, n (%)	6 (6.9)	87 (5.7)	0.651
AKI stage 2, n (%)	4 (4.6)	15 (1.0)	0.018
AKI stage 3, n (%)	10 (11.5)	28 (1.8)	<0.001
New permanent haemodialysis, n (%)	2 (2.3)	9 (0.6)	0.132
Conversion to open surgery, n (%)	0 (0.0)	20 (1.3)	0.638
Cardiac tamponade, n (%)	1 (1.2)	25 (1.6)	0.711
Valve embolisation, n (%)	1 (1.2)	8 (0.5)	0.502
Second valve, n (%)	2 (2.3)	19 (1.3)	0.444
Worsened CHF after TAVR before discharge, n (%)	12 (16.9)	56 (4.3)	<0.001
Postoperative echocardiographic data			
LVEF (modified Simpson), %	51.3±13.4	58.2±11.3	<0.001
Index effective orifice area, cm^2^/m^2^	1.2±0.3	1.2±0.3	0.375
Mean pressure gradient, mm Hg	10.2±3.9	10.2±3.9	0.953
Aortic regurgitation ≥ moderate, n (%)	2 (2.4)	15 (1.0)	0.295
Mitral regurgitation ≥ moderate, n (%)	13 (15.5)	89 (5.9)	0.003
Tricuspid regurgitation ≥ moderate, n (%)	11 (13.8)	97 (6.8)	0.035

Values are presented as mean±SD deviation unless otherwise stated.

P<0.05 were considered statistically significant.

AKI, acute kidney injury; CHF, congestive heart failure; ECMO, extracorporeal membrane oxygenation; El-TAVR, elective TAVR; Em-TAVR, urgent/emergent/salvage TAVR; LVEF, left ventricular ejection fraction; TAVR, transcatheter aortic valve replacement.

**Table 3:** The multivariate logistic regression analysis for predictors for the need for urgent/emergent/salvage transcatheter aortic valve replacement

Patient characteristics	Univariate analysis		Multivariate analysis	
	OR (95% CI)	P value	OR (95% CI)	P value
BMI (per 1.0 kg/m^2^ increase)	0.92 (0.87 to 0.99)	0.017	0.93 (0.87 to −1.01)	0.084
Clinical Frailty Scale (per 1.0 category)	1.78 (1.52 to 2.10)	<0.001	1.50 (1.24 to 1.83)	<0.001
PAD	2.68 (1.66 to 4.32)	<0.001	2.34 (1.32 to 4.13)	0.003
Haemoglobin (per 1.0 g/dL increase)	0.83 (0.73 to 0.96)	0.010	0.94 (0.79 to 1.11)	0.475
Albumin <3.5 g/dL	4.17 (2.66 to 6.52)	<0.001	2.15 (1.25 to 3.68)	0.006
eGFR (per 1.0 mL/min/1.73 m^2^ increase)	0.98 (0.97 to 0.99)	0.002	0.99 (0.98 to 1.00)	0.089
Atrial fibrillation	2.19 (1.39 to 3.45)	0.001	1.26 (0.72 to 2.21)	0.412
LVEF (per 1.0% increase)	0.94 (0.93 to 0.96)	<0.001	0.96 (0.94 to 0.97)	<0.001
AVA (per 0.1 cm2 increase)	0.75 (0.65 to 0.86)	<0.001	0.88 (0.75 to 1.02)	0.094
MR ≥ moderate	3.83 (2.32 to 6.33)	<0.001	2.60 (1.42 to 4.74)	0.002
TR ≥ moderate	2.74 (1.46 to 5.12)	0.004	1.48 (0.64 to 3.42)	0.355

P<0.05 were considered statistically significant.

BMI, body mass index; PAD, peripheral artery disease; eGFR, estimated glomerular filtration rate; LVEF, left ventricular ejection fraction; AVA, aortic valve area; MR, mitral regurgitation; TR, tricuspid regurgitation.

### Cumulative survival rate of the urgency of TAVR

**Figure 1:**
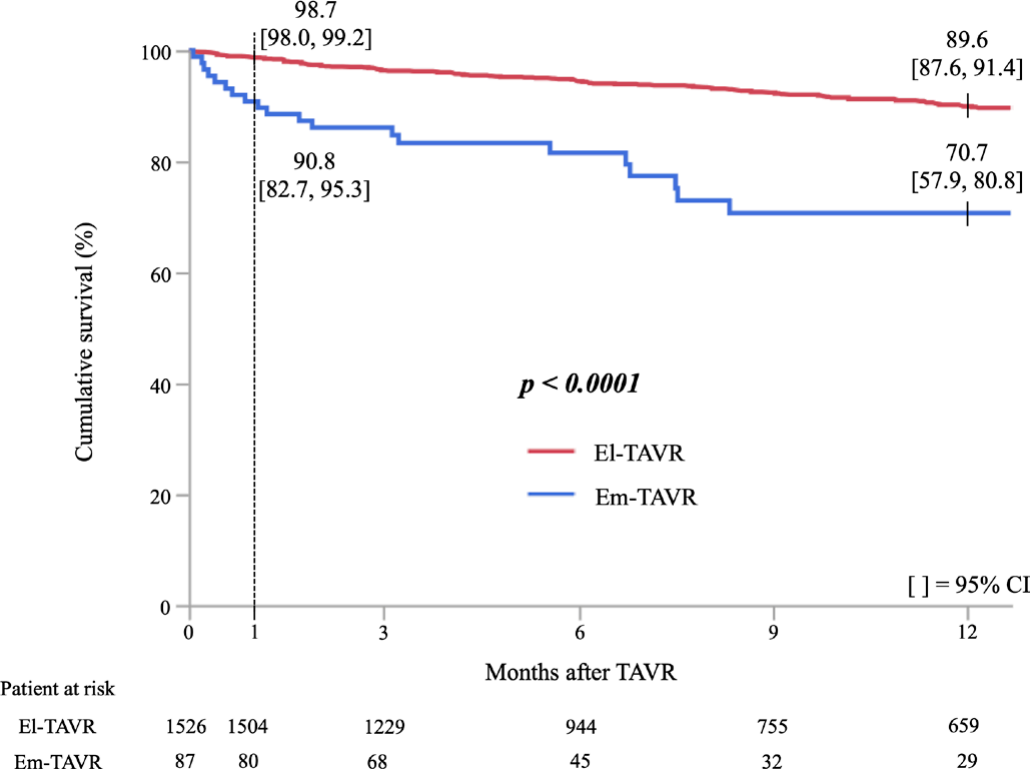
Cumulative survival curves according to urgency of transcatheter aortic valve replacement (TAVR). El-TAVR=elective TAVR; Em-TAVR=urgent/emergent/salvage TAVR.

**Figure 2:**
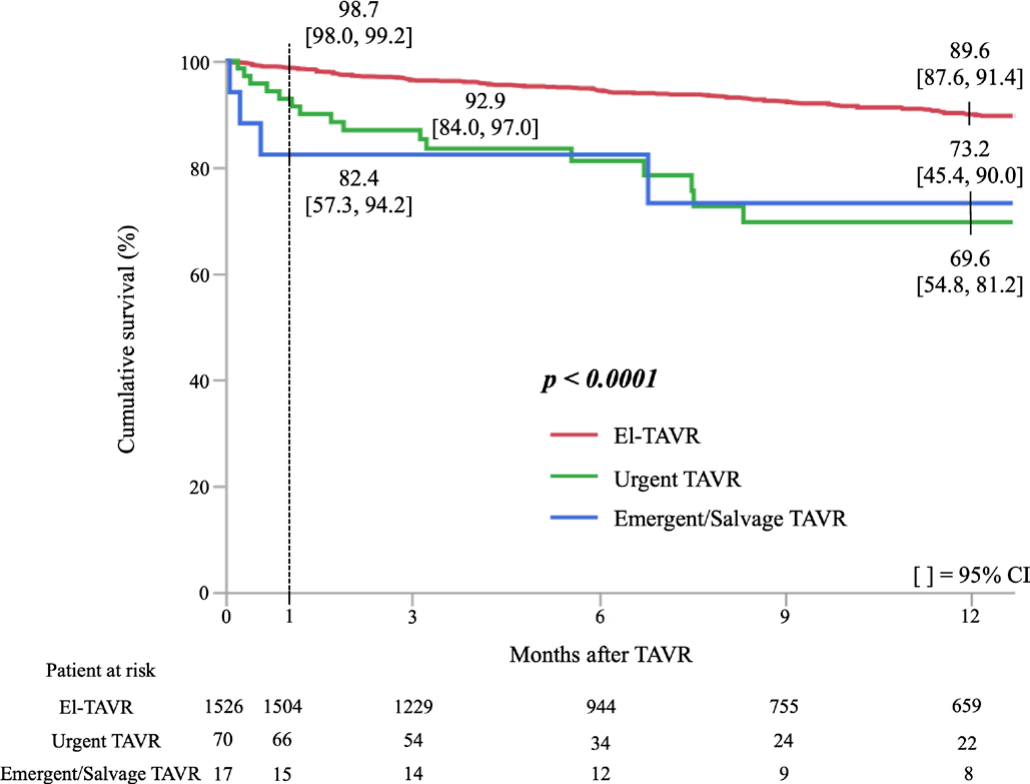
Cumulative survival curves according to urgency of transcatheter aortic valve replacement (TAVR). El-TAVR=elective TAVR.

The cumulative survival rate in Kaplan-Meier analysis was significantly lower in Em-TAVR patients compared with El-TAVR patients (log-rank test; p<0.001) (figures 1 and 2). There was a significant difference in the cumulative survival rate between El-TAVR and urgent TAVR patients (log-rank test; p<0.001) (online supplemental figure 1). However, the cumulative survival did not differ between urgent and emergent/salvage TAVR patients (log-rank test; p=0.996) (online supplemental figure 2).

10.1136/openhrt-2020-001467.supp2 Supplementary data

10.1136/openhrt-2020-001467.supp3 Supplementary data

### Subgroup analysis in the Em-TAVR patient cohort

We performed a subgroup analysis to investigate variables associated with 1-year mortality in Em-TAVR patients. The Em-TAVR patients were divided into two groups based on whether or not they had survived for 1 year after TAVR; 20/87 (23.0%) had died with 1 year of TAVR. The baseline patient characteristics of these patients are presented in online supplemental table 2). Compared with the survivor group, the CFS Score was higher (5.6±1.4 vs 4.8±1.2; p=0.016) and haemoglobin level (9.6±1.6 g/dL vs 11.1±1.8 g/dL, p=0.002) was lower in the mortality group. The prevalence of prior coronary artery bypass graft (CABG) (25.0% vs 6.0%; p=0.024) and prior stroke (40.0% vs 17.9%; p=0.048) in the mortality group was higher than in the survivor group. The procedural characteristics, clinical outcomes and procedural complications among the two groups are presented in online supplemental table 3. Seventeen patients in the mortality group underwent postoperative echocardiography. The prevalence of at least moderate aortic regurgitation (11.8% vs 0.0%; p=0.010) and at least moderate TR (29.4% vs 9.0%; p=0.026) was higher in the mortality group than in the survivor group. In the univariate logistic regression analysis, a higher CFS (OR 1.50, 95% CI 1.06 to 2.14, p=0.023), prior CABG (OR 3.18, 95% CI 1.15 to 8.77, p=0.026), lower serum haemoglobin concentration (OR 0.71, 95% CI 0.54 to 0.91, p=0.010) and lower serum albumin concentration (OR 0.91, 95% CI 0.85 to 0.98, p=0.014) were significantly associated with an increased risk of 1-year mortality after Em-TAVR (online supplemental table 4).

## Discussion

We made three important clinical observations in this study. First, the percentage of patients with severe AS who needed to undergo Em-TAVR in our study was 5.4% (87/1613). Second, the predictors for the need for Em-TAVR were a high CFS, a history of PAD, hypoalbuminaemia, reduced LVEF and at least moderate MR. Although Kaplan-Meier analysis revealed that cumulative mortality was higher in Em-TAVR patients than in El-TAVR patients, the Cox regression analysis revealed that Em-TAVR was not associated with cumulative mortality after TAVR. To the best of our knowledge, this is the first report on Em-TAVR from an Asian multicentre registry.

The authors summarised the principal findings on Em-TAVR obtained from this study, the study from the STS/ACC TVT Registry^4^ and the study by Frerker *et al*,^5^ detailing the patients’ characteristics, the prevalence of Em-TAVR, the acute device success rate of the TAVR procedure and the mortality rate in online supplemental table 5. Compared with the study from the STS/ACC TVT Registry, our study included patients with a higher Surgical Risk Score and showed similar mortality rates after Em-TAVR.^4^ As expected, the baseline conditions of patients with Em-TAVR were significantly worse than those with El-TAVR in our study. Although the cumulative mortality after Em-TAVR was higher than after El-TAVR, the clinical outcomes in Em-TAVR seemed to be acceptable in our study. Further, urgency did not negatively affect the mortality after TAVR (online supplemental table 1). Considering these findings, we believe that there is no need for hesitation when deciding to perform Em-TAVR. Prolonged hospitalisation due to observation treatment is inherently harmful to older patients because bed rest is associated with sarcopenia, infections and a greater length of stay.^11^ However, early mobilisation due to early intervention may result in a reduced length of stay and reduce the complications associated with bed rest. Thus, it may be better to plan the procedure ahead of time for patients with severe AS with congestive heart failure, especially in those using catecholamines and/or MCS. Although balloon aortic valvuloplasty (BAV) can be another treatment option in an emergent setting, Bongiovanni *et al* showed that El-TAVR following to emergent BAV was not superior to Em-TAVR without emergent BAV in terms of survival.^6^ BAV may be considered when TAVR is inappropriate due to anatomical problems.

This study showed that the predictors for the need for Em-TAVR were higher CFS, a history of PAD, hypoalbuminaemia, reduced LVEF and at least moderate preoperative MR (table 3). Although previous studies have shown that these factors are predictors of mortality after TAVR,^12–20^ it was unknown whether they were also the predictors for the need for Em-TAVR. CFS, hypoalbuminaemia and PAD have been accepted as a general indicators of a patient’s vulnerability and are highly associated with adverse health outcomes in the geriatric field.^21–24^ Our study showed that preoperative moderate or severe MR was associated with Em-TAVR. In both SAVR and TAVR, previous studies have shown a higher mortality following TAVR in patients with significant MR than in those without.^17–20^ Moreover, higher CFS, prior CABG, lower serum haemoglobin concentration and lower serum albumin concentration were associated with 1-year mortality after Em-TAVR (online supplemental table 4). The CFS, serum haemoglobin concentration and serum albumin concentration in our study were associated with the need for Em-TAVR and 1-year mortality after Em-TAVR. High CFS and low haemoglobin and albumin concentration level, together with, reduced LVEF or significant MR, could be signs encouraging clinicians to conduct TAVR earlier.

Regarding study limitations, a selection bias may exist in our study because the decision regarding the performance of Em-TAVR was at the discretion of the local heart team and the details of the reason for Em-TAVR were unknown. Due to the small number of the patients who underwent Em-TAVR, we could not perform multivariate analysis to identify independent predictors for 1-year mortality after Em-TAVR.

## Conclusion

Em-TAVR was observed in 5.4% of patients in this study. The predictors for the need for Em-TAVR were identified as a higher CFS, PAD, hypoalbuminaemia, reduced LVEF and at least moderate MR. The multivariate Cox regression analysis revealed that Em-TAVR was not associated with cumulative mortality after TAVR, suggesting that Em-TAVR is a safe and reasonable treatment option.

## References

[1] Nishimura RA, Otto CM, Bonow RO, et al 2017 AHA/ACC Focused Update of the 2014 AHA/ACC Guideline for the Management of Patients With Valvular Heart Disease: A Report of the American College of Cardiology/American Heart Association Task Force on Clinical Practice Guidelines. J Am Coll Cardiol 2017;70:252–89. 10.1016/j.jacc.2017.03.011 28315732

[2] Mack MJ, Leon MB, Thourani VH, et al Transcatheter aortic-valve replacement with a Balloon-Expandable valve in low-risk patients. N Engl J Med 2019;380:1695–705. 10.1056/NEJMoa1814052 30883058

[3] Popma JJ, Deeb GM, Yakubov SJ, et al Transcatheter aortic-valve replacement with a self-expanding valve in low-risk patients. N Engl J Med 2019;380:1706–15. 10.1056/NEJMoa1816885 30883053

[4] Kolte D, Khera S, Vemulapalli S, et al Outcomes following Urgent/Emergent transcatheter aortic valve replacement: insights from the STS/ACC TVT registry. JACC Cardiovasc Interv 2018;11:1175–85. 10.1016/j.jcin.2018.03.002 29929641

[5] Frerker C, Schewel J, Schlüter M, et al Emergency transcatheter aortic valve replacement in patients with cardiogenic shock due to acutely decompensated aortic stenosis. EuroIntervention 2016;11:1530–6. 10.4244/EIJY15M03_03 25751886

[6] Bongiovanni D, Kühl C, Bleiziffer S, et al Emergency treatment of decompensated aortic stenosis. Heart 2018;104:23–9. 10.1136/heartjnl-2016-311037 28566471

[7] Landes U, Orvin K, Codner P, et al Urgent transcatheter aortic valve implantation in patients with severe aortic stenosis and acute heart failure: procedural and 30-day outcomes. Can J Cardiol 2016;32:726–31. 10.1016/j.cjca.2015.08.022 26755244

[8] Yamamoto M, Watanabe Y, Tada N, et al Transcatheter aortic valve replacement outcomes in Japan: optimized catheter vAlvular iNtervention (Ocean) Japanese multicenter registry. Cardiovasc Revasc Med 2019;20:843-851. 10.1016/j.carrev.2018.11.024 30553819

[9] Nashef SAM, Roques F, Sharples LD, et al EuroSCORE II. Eur J Cardiothorac Surg 2012;41:734–45. 10.1093/ejcts/ezs043 22378855

[10] Kappetein AP, Head SJ, Généreux P, et al Updated standardized endpoint definitions for transcatheter aortic valve implantation: the valve academic research Consortium-2 consensus document (VARC-2). Eur J Cardiothorac Surg 2012;42:S45–60. 10.1093/ejcts/ezs533 23026738

[11] Surkan MJ, Gibson W Interventions to mobilize elderly patients and reduce length of hospital stay. Can J Cardiol 2018;34:881–8. 10.1016/j.cjca.2018.04.033 29960617

[12] Shimura T, Yamamoto M, Kano S, et al Impact of the clinical frailty scale on outcomes after transcatheter aortic valve replacement. Circulation 2017;135:2013–24. 10.1161/CIRCULATIONAHA.116.025630 28302751

[13] Fanaroff AC, Manandhar P, Holmes DR, et al Peripheral artery disease and transcatheter aortic valve replacement outcomes: a report from the Society of thoracic Surgeons/American College of cardiology transcatheter therapy registry. Circ Cardiovasc Interv 2017;10. 10.1161/CIRCINTERVENTIONS.117.005456 PMC5683430 29042398

[14] Yamamoto M, Shimura T, Kano S, et al Prognostic value of hypoalbuminemia after transcatheter aortic valve implantation (from the Japanese multicenter OCEAN-TAVI registry). Am J Cardiol 2017;119:770–7. 10.1016/j.amjcard.2016.11.019 28017301

[15] Gassa A, Borghardt JH, Maier J, et al Effect of preoperative low serum albumin on postoperative complications and early mortality in patients undergoing transcatheter aortic valve replacement. J Thorac Dis 2018;10:6763–70. 10.21037/jtd.2018.11.30 30746221 PMC6344723

[16] Gotzmann M, Rahlmann P, Hehnen T, et al Heart failure in severe aortic valve stenosis: prognostic impact of left ventricular ejection fraction and mean gradient on outcome after transcatheter aortic valve implantation. Eur J Heart Fail 2012;14:1155–62. 10.1093/eurjhf/hfs108 22782969

[17] Sannino A, Losi MA, Schiattarella GG, et al Meta-Analysis of mortality outcomes and mitral regurgitation evolution in 4,839 patients having transcatheter aortic valve implantation for severe aortic stenosis. Am J Cardiol 2014;114:875–82. 10.1016/j.amjcard.2014.06.022 25092192

[18] Nombela-Franco L, Eltchaninoff H, Zahn R, et al Clinical impact and evolution of mitral regurgitation following transcatheter aortic valve replacement: a meta-analysis. Heart 2015;101:1395–405. 10.1136/heartjnl-2014-307120 26060121

[19] Toggweiler S, Boone RH, Rodés-Cabau J, et al Transcatheter aortic valve replacement: outcomes of patients with moderate or severe mitral regurgitation. J Am Coll Cardiol 2012;59:2068–74. 10.1016/j.jacc.2012.02.020 22483326

[20] D'Onofrio A, Gasparetto V, Napodano M, et al Impact of preoperative mitral valve regurgitation on outcomes after transcatheter aortic valve implantation. Eur J Cardiothorac Surg 2012;41:1271–7. 10.1093/ejcts/ezr236 22219481

[21] Fried LP, Tangen CM, Walston J, et al Frailty in older adults: evidence for a phenotype. J Gerontol A Biol Sci Med Sci 2001;56:M146–57. 10.1093/gerona/56.3.M146 11253156

[22] Gopal DM, Kalogeropoulos AP, Georgiopoulou VV, et al Serum albumin concentration and heart failure risk the health, aging, and body composition study. Am Heart J 2010;160:279–85. 10.1016/j.ahj.2010.05.022 20691833 PMC2919495

[23] Kempny A, Diller G-P, Alonso-Gonzalez R, et al Hypoalbuminaemia predicts outcome in adult patients with congenital heart disease. Heart 2015;101:699–705. 10.1136/heartjnl-2014-306970 25736048 PMC4413739

[24] Miller AP, Huff CM, Roubin GS Vascular disease in the older adult. J Geriatr Cardiol 2016;13:727–32. 10.11909/j.issn.1671-5411.2016.09.011 27899936 PMC5122497

