# Supplementary material for: Patients’ characteristics and mortality in urgent/emergent/salvage transcatheter aortic valve replacement: insight from the OCEAN-TAVI registry
Source: Open Heart. 2020 Dec 14;7(2):e001467. doi: 10.1136/openhrt-2020-001467 (PMC7737081; doi:10.1136/openhrt-2020-001467)
Supplement: Supplementary data [file openhrt-2020-001467supp001.pdf]

Supplementary Table 1. The multivariate Cox regression analysis for mortality after transcatheter aortic valve replacement.

Background	Univariate analysis		Multivariate analysis	
	**HR (95% CI)**	**P-value**	**Adjusted HR (95% CI)**	**P-value**
Em-TAVR	3.36 (2.14–5.26)	< 0.001	1.63 (0.95–2.81)	0.076
Age (per 1-year-old increase)	1.01 (0.98–1.04)	0.392	1.06 (1.03–1.10)	< 0.001
BMI (per 1.0 kg/m^2 increase)	0.95 (0.91–1.00)	0.032	0.96 (0.91–1.01)	0.166
Clinical frailty scale score (per 1 category increase)	1.41 (1.26–1.59)	< 0.001	1.32 (1.15–1.51)	< 0.001
PAD	1.82 (1.28–2.59)	< 0.001	1.33 (0.89-1.99)	0.167
Atrial fibrillation	1.77 (1.26-2.47)	< 0.001	1.23 (0.84–2.82)	0.293
Hemoglobin (per 1 g/dl increase)	0.74 (0.67–0.81)	< 0.001	0.83 (0.74–0.93)	0.002
Albumin <3.5 g/dl	2.68 (1.97–3.64)	< 0.001	1.77 (1.23–2.56)	0.003
eGFR (per 1.0 ml/min/1.73 m^2 increase)	0.98 (0.97–0.99)	< 0.001	0.99 (0.98–1.00)	0.009
LVEF (per 1.0% increase)	0.99 (0.98–1.01)	0.426	1.01 (0.99–1.02)	0.293

P-values <0.05 were considered statistically significant.

HR = hazard ratio; CI = confidence interval; Em-TAVR = urgent/emergent/salvage transcatheter aortic valve replacement; BMI = body mass index; PAD = peripheral artery disease; eGFR = estimated glomerular filtration rate; LVEF = left ventricular ejection fraction

Supplementary Table 2. Baseline patient characteristics of urgent/emergent/salvage transcatheter aortic valve replacement in patients with or without mortality

Variable	Mortality group (n = 20 [23.0%])	Survivor group (n = 67 [77.0%])	P-value
**Baseline patient characteristic**			
Age, years	84.4 ± 8.9	85.1 ± 6.4	0.702
Female, n (%)	11 (55.0)	50 (74.6)	0.101
Height, cm	150.5 ± 9.5	148.7 ± 7.9	0.415
Weight, kg	46.8 ± 11.5	47.5 ± 8.7	0.783
Body mass index, kg/m^2	20.8 ± 3.9	21.4 ± 3.2	0.451
Clinical frailty scale score	5.6 ± 1.4	4.8 ± 1.2	0.016
NYHA functional class Ⅲ or Ⅳ, n (%)	17 (85.0)	60 (89.6)	0.586
Prior heart failure, n (%)	20 (100.0)	61 (91.0)	0.071
Syncope, n (%)	3 (15.0)	10 (14.9)	0.993
Current smoker, n (%)	3 (15.0)	4 (6.0)	0.222
Hypertension, n (%)	15 (75.0)	50 (74.6)	0.973
Diabetes mellitus, n (%)	7 (35.0)	22 (32.8)	0.857
Dyslipidemia, n (%)	10 (50.0)	30 (44.8)	0.681
Peripheral artery disease, n (%)	8 (40.0)	19 (28.4)	0.331
COPD, n (%)	4 (20.0)	11 (16.4)	0.714
Atrial fibrillation, n (%)	7 (35.0)	24 (35.8)	0.946
Prior MI, n (%)	4 (20.0)	9 (13.4)	0.482
Prior PCI, n (%)	8 (40.0)	19 (28.4)	0.331
Prior CABG, n (%)	5 (25.0)	4 (6.0)	0.024
Prior pacemaker implantation, n (%)	1 (5.0)	7 (10.5)	0.432
Prior stroke, n (%)	8 (40.0)	12 (17.9)	0.048
Urgency of procedure			0.708
Elective, n (%)	0 (0.0)	0 (0.0)	
Urgent without catecholamine or MCS, n (%)	8 (40.0)	33 (49.3)	

Urgent with catecholamine or MCS, n (%)	8 (40.0)	21 (31.3)	
Emergent, n (%)	3 (15.0)	12 (17.9)	
Salvage, n (%)	1 (5.0)	1 (1.5)	
STS score, %	13.9 (8.7-20.1)	13.7 (7.9-22.5)	0.904
Logistic Euro SCORE, %	40.5 (27.4-52.2)	24.5 (13.9-46.9)	0.014
EuroSCORE II, %	14.0 (8.3-29.2)	9.9 (5.9-16.9)	0.057
State of catecholamine dependency, n (%)	10 (50.0)	26 (38.8)	0.375
Use of IABP, n (%)	4 (20.0)	4 (6.0)	0.058
**Laboratory data**			
Hemoglobin concentration, g/dl	9.6 ± 1.6	11.1 ± 1.8	0.002
eGFR, ml/min/1.73 m^2	40.9 ± 25.0	46.8 ± 21.2	0.293
Albumin, g/dl	3.1 ± 0.6	3.5 ± 0.5	0.005
Albumin <3.5 g/dl, n (%)	15 (75.0)	39 (58.2)	0.165
Brain natriuretic peptide, pg/ml	1401 ± 1864	1139 ± 1172	0.466
**Preoperative echocardiographic data**			
LVEF (modified Simpson), %	48.1 ± 18.9	47.9 ± 15.4	0.963
Bicuspid valve, n (%)	0 (0.0)	0 (0)	-
Aortic valve area, cm2	0.58 ± 0.16	0.55 ± 0.15	0.573
Index aortic valve area, cm2/m2	0.42 ± 0.12	0.40 ± 0.11	0.644
Mean pressure gradient, mmHg	45.6 ± 21.8	51.6 ± 19.4	0.239
Peak velocity, m/s	4.2 ± 0.99	4.6 ± 0.78	0.103
Aortic regurgitation ≥moderate, n (%)	1 (5.0)	8 (11.9)	0.337
Mitral regurgitation ≥ moderate, n (%)	8 (40.0)	16 (23.9)	0.167
Tricuspid regurgitation ≥ moderate, n (%)	5 (25.0)	8 (11.9)	0.171

Values are presented as mean ± standard deviation unless otherwise stated.

P-values <0.05 were considered statistically significant.

NYHA = New York Heart Association; COPD = chronic obstructive pulmonary disease; MI = myocardial infarction; PCI = percutaneous coronary intervention; CABG = coronary artery bypass graft; MCS =; mechanical circulatory support; STS =: Society of Thoracic Surgeons;

EuroSCORE = European System for Cardiac Operative Risk Evaluation; IABP = intra-aortic balloon pumping; eGFR = estimated glomerular filtration rate; LVEF =: left ventricular ejection fraction.

Supplementary Table 3. Procedural characteristics and clinical outcomes of urgent/emergent/salvage transcatheter aortic valve replacement in patients with or without mortality

Variable	Mortality group (n = 20 [23.0%])	Survivor group (n=67 [77.0%])	P-value
**Procedural characteristics**			
Transfemoral approach, n (%)	16 (80.0)	55 (82.1)	0.834
Bioprosthetic valve type			0.826
Sapien XT, n (%)	17 (85.0)	53 (79.1)	
Sapien 3, n (%)	1 (5.0)	4 (6.0)	
CoreValve, n (%)	2 (10.0)	10 (14.9)	
Predilatation, n (%)	15 (75.0)	50 (74.6)	0.973
Postdilatation, n (%)	4 (20.0)	14 (20.9)	0.780
Use of ECMO, n (%)	6 (30.0)	8 (11.9)	0.068
Elective ECMO, n (%)	4 (20.0)	6 (9.0)	0.276
Emergent ECMO, n (%)	2 (10.0)	2 (3.0)	0.227
Contrast volume, ml	138.8 ± 92.0	115.4 ± 63.9	0.207
Fluoroscope time, min	31.2 ± 17.2	23.3 ± 9.4	0.010
**Clinical outcomes and complications**			
30-day mortality, n (%)	8 (40.0)	0 (0.0)	< 0.001
In-hospital death, n (%)	10 (50.0)	0 (0.0)	< 0.001
Device success, n (%)	12 (60.0)	64 (95.5)	< 0.001
Acute coronary obstruction, n (%)	0 (0.0)	1 (1.5)	0.468
New pacemaker implantation, n (%)	1 (5.0)	7 (10.5)	0.432
Stroke, n (%)	2 (10.0)	1 (1.5)	0.100
Life-threatening or disabling bleeding, n (%)	8 (40.0)	5 (7.5)	0.001
Major bleeding, n (%)	4 (20.0)	15 (22.4)	0.819
Transfusion, n (%)	13 (65.0)	34 (50.8)	0.258
Major vascular complication, n (%)	4 (20.0)	9 (13.4)	0.482
AKI stage 1, n (%)	1 (5.0)	5 (7.5)	0.693
AKI stage 2, n (%)	2 (10.0)	2 (3.0)	0.227
AKI stage 3, n (%)	5 (25.0)	5 (7.5)	0.045

New permanent hemodialysis, n (%)	1 (5.0)	1 (1.5)	0.400
Conversion to open surgery, n (%)	0 (0.0)	0 (0.0)	-
Cardiac tamponade, n (%)	1 (5.0)	0 (0.0)	0.084
Valve embolization, n (%)	1 (4.6)	0 (0.0)	0.084
Second valve, n (%)	0 (0)	2 (3.0)	0.304
Worsened CHF after TAVR before discharge, n (%)	7 (35.0)	10 (14.9)	0.058
**Postoperative echocardiographic data**			
LVEF (modified Simpson), %	50.9 ± 15.9	51.3 ± 13.1	0.918
Index effective orifice area, cm^2/m^2	1.1 ± 0.2	1.2 ± 0.3	0.281
Mean pressure gradient, mmHg	9.9 ± 3.5	10.3 ± 4.0	0.742
Aortic regurgitation ≥moderate, n (%)	2 (11.8)	0 (0.0)	0.010
Mitral regurgitation ≥moderate, n (%)	3 (17.7)	10 (14.9)	0.785
Tricuspid regurgitation ≥moderate, n (%)	5 (29.4)	6 (9.0)	0.026

Values are presented as mean ± standard deviation unless otherwise stated.

P-values <0.05 were considered statistically significant.

ECMO = extracorporeal membrane oxygenation; CHF = congestive heart failure; AKI = acute kidney injury; TAVR = transcatheter aortic valve replacement; LVEF = left ventricular ejection fraction.

Supplementary Table 4. The univariate logistic regression analysis for risk factors for 1-year mortality in patients with undergoing urgent/emergent/salvage transcatheter aortic valve replacement.

**Background**	**Univariate analysis**	
	**OR (95% CI)**	**P-value**
Age (per 1-year-old increase)	0.98 (0.92-1.05)	0.576
BMI (per 1.0 kg/m^2 increase)	0.96 (1.10-1.04)	0.567
Clinical frailty scale (per 1 category increase)	1.50 (1.06–2.14)	0.023
PAD	1.81 (0.74-4.46)	0.195
Atrial fibrillation	0.94 (0.37-2.32)	0.867
Prior CABG	3.18 (1.15-8.77)	0.026
Prior stroke	2.43 (0.99-5.94)	0.052
Hemoglobin (per 1.0 g/dl increase)	0.71 (0.54–0.91)	0.010
Albumin (per 0.1 g/dl increase)	0.91 (0.85–0.98)	0.014
eGFR (per 1.0 ml/min/1.73 m^2 increase)	0.99 (0.96-1.01)	0.292
LVEF (per 1.0% increase)	1.00 (0.97-1.03)	0.950

P-values <0.05 were considered statistically significant.

OR = odds ratio; CI = confidence interval; BMI = body mass index; PAD = peripheral artery disease; CABG = coronary artery bypass graft; eGFR = estimated glomerular filtration rate; LVEF = left ventricular ejection fraction.

Supplementary Table 5. The baseline characteristics and outcomes in the current study and previous studies.

Author	Our study (n = 1613)	The STS/ACC TVT Registry, Kolte et al.[4] (n = 40,042)	Christian Frerker et al.[5] (n = 771)
Number of urgent/emergent/salvage TAVR, n (%)	87 (5.4)	3,952 (9.9)	27 (3.5)
Urgent, n (%)	70 (4.3)	3,888 (9.7)	
Emergent, n (%)	15 (0.9)	64 (0.2)	27 (3.5)
Salvage, n (%)	2 (0.1)	0 (0.0)	
STS score, %	13.7 (8.2-21.0)	11.8 (7.6-17.9)	-
Logistic EuroSCORE, %	35.1 ± 22.0	-	60.4 ± 21.1
Device success, %	87.4	92.6	88.9
30-day mortality, %	9.2	8.7	33.3
1-year mortality, %	23.0	29.1	40.7

STS = Society of Thoracic Surgeons; ACC = American College of Cardiology; TVT = Transcatheter Valve Therapy; TAVR = transcatheter aortic valve replacement; EuroSCORE = European System for Cardiac Operative Risk Evaluation.
