# Supplementary figures and images for: Patients’ characteristics and mortality in urgent/emergent/salvage transcatheter aortic valve replacement: insight from the OCEAN-TAVI registry
Source: Open Heart. 2020 Dec 14;7(2):e001467. doi: 10.1136/openhrt-2020-001467 (PMC7737081; doi:10.1136/openhrt-2020-001467)

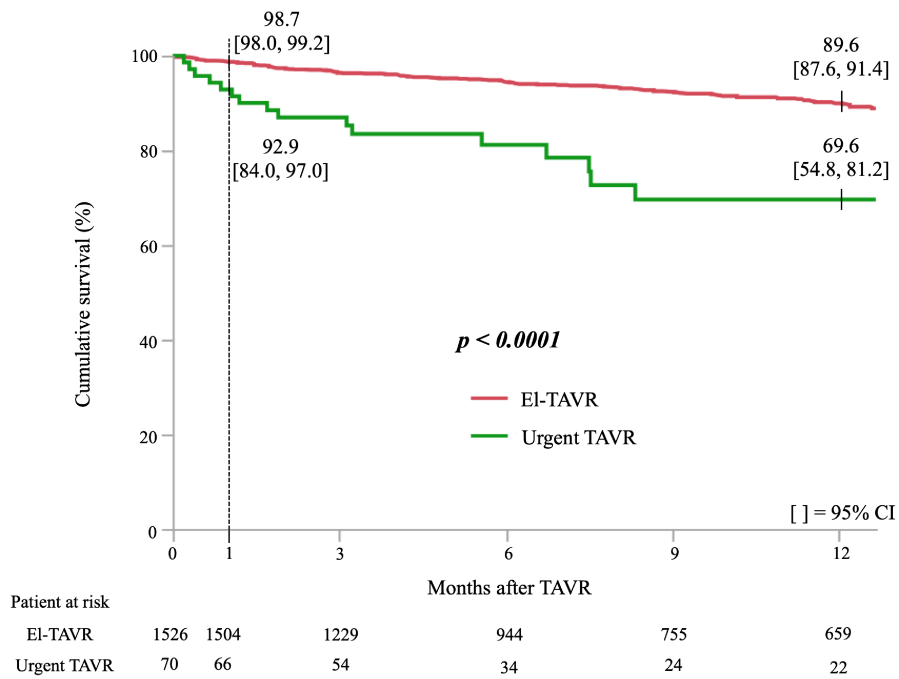

Supplement: Supplementary data [file openhrt-2020-001467supp002.pdf]

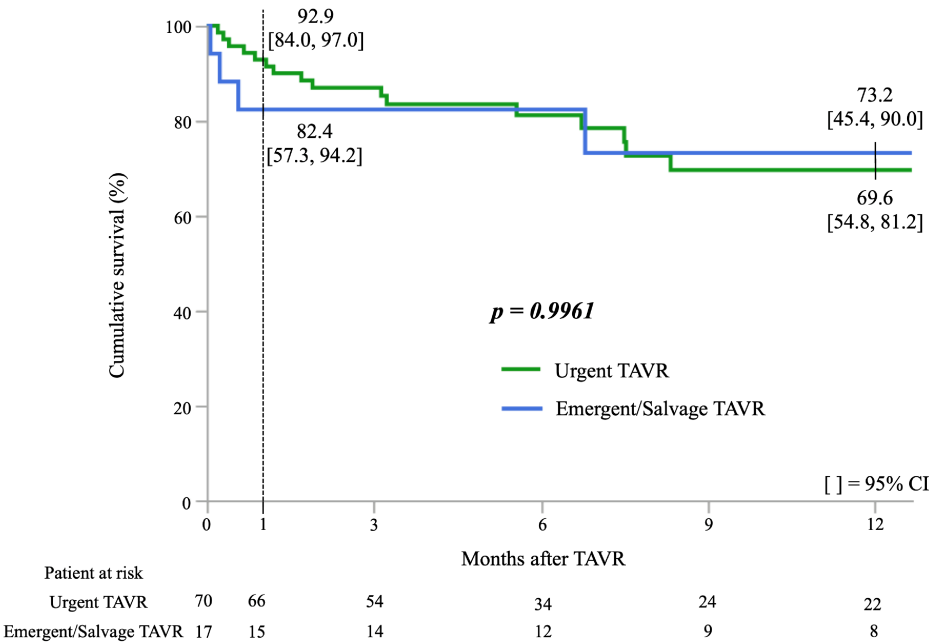

Supplement: Supplementary data [file openhrt-2020-001467supp003.pdf]
